# 160. Antimicrobial Prescribing Patterns in Bloodstream Infections Caused by Cefepime-Susceptible Extended Spectrum β-lactamase Enterobacterales

**DOI:** 10.1093/ofid/ofac492.238

**Published:** 2022-12-15

**Authors:** Emerald O’Rourke, Amanda Binkley, Naasha J Talati, Sonal Patel, Christina Maguire

**Affiliations:** Penn Presbyterian Medical Center, Philadelphia, Pennsylvania; Penn Presbyterian Medical Center, Philadelphia, Pennsylvania; Penn Presbyterian Medical Center, Philadelphia, Pennsylvania; Hospital of the University of Pennsylvania, Philadelphia, Pennsylvania; Penn Presbyterian Medical Center, Philadelphia, Pennsylvania

## Abstract

**Background:**

The reliability of piperacillin-tazobactam (TZP) and cefepime (FEP) breakpoints for extended spectrum β-lactamase Enterobacterales (ESBL-E) isolates remains controversial. The Infectious Diseases Society of America recommends against the use of TZP and FEP for the treatment of bloodstream infections (BSIs) caused by ESBL-E, even when in-vitro susceptibility is demonstrated. The University of Pennsylvania Health System microbiology laboratory suppresses TZP susceptibilities on Escherichia coli and Klebsiella pneumoniae blood isolates nonsusceptible to ceftriaxone (CRO) or ceftazidime (CAZ) and specifies the presence of a multidrug resistant organism. However, FEP susceptibilities are reported. The objective of this study was to assess appropriate antimicrobial prescribing for the treatment of FEP-susceptible ESBL-E BSIs with our institution’s current susceptibility reporting practices.

**Methods:**

This multicenter retrospective observational study included patients with blood cultures positive for E. coli, K. oxytoca, K. pneumoniae, and Proteus mirabilis nonsusceptible to CRO or CAZ but susceptible to FEP from August 1, 2018 through August 1, 2021. Patients with a concominant gram-negative infection or true beta-lactam allergy were excluded. Patients were assessed for appropriate therapy (demonstrated susceptibility to and administration of carbapenems fluoroquinolones, novel beta-lactam/beta-lactamase inhibitors, or sulfamethoxazole-trimethoprim) within 24 h following the availability of susceptibility results.

**Results:**

During the study period 38 patients were included. Of the 38 patients, 52.6% (n=20) received appropriate therapy with an average time to appropriate therapy of 3.7 (SD 2.1) h. Among patients receiving inappropriate therapy (n=18), 50% (n=9) were changed to appropriate therapy >24 h after susceptibility results, with an average time to appropriate therapy of 46.5 (SD 44.4) h. The remaining 9 patients (23.7%) did not receive appropriate therapy beyond 24 h.

**Conclusion:**

Reporting FEP susceptibility results on ESBL-E blood isolates may contribute to the prescribing of FEP for the treatment of ESBL-E BSIs and delay appropriate antimicrobial therapy. Given these findings, cefepime will now be suppressed on ESBL-E blood isolates.

**Disclosures:**

**All Authors**: No reported disclosures.

